# The correlation between Flt3-ITD mutation in dendritic cells with TIM-3 expression in acute myeloid leukemia

**DOI:** 10.1097/BS9.0000000000000092

**Published:** 2021-10-18

**Authors:** Hooriyeh Shapoorian, Hamidreza Zalpoor, Mazdak Ganjalikhani-Hakemi

**Affiliations:** a Department of Immunology, School of Medicine, Isfahan University of Medical Sciences, Isfahan, Iran; b Paramedical School, Shiraz University of Medical Sciences, Shiraz, Iran; c Acquired Immunodeficiency Research Center, Isfahan University of Medical Sciences, Isfahan, Iran.

**Keywords:** Acute myeloid leukemia, Dendritic cell, Flt3-ITD, TIM-3

## Abstract

In general, acute myeloid leukemia (AML) is an aggressive and heterogeneous disease that is characterized by rapid cellular proliferation and high mortality. One of the mutations related to AML is the Flt3-ITD mutation, which is found in approximately 25% of patients. In this mini-review, we investigate the function of dendritic cells and T cells based on Flt3-ITD mutation and immune evasion as a result of this abnormality. Finally, we discuss some AML therapeutic strategies, including targeting Flt3 on DCs and TIM-3 on T cells as immune receptors to treat this hematopoietic malignancy.

## 1 INTRODUCTION

Acute myeloid leukemia (AML) is a type of cancer that manifests clonal cell proliferation, abnormal differentiation, and poor differentiation of hematopoietic cells inside the bone marrow, blood, and other tissues.^1^ Patients who are difficult to treat are mainly older adults. As a result, overall survival over 5 years is only about 27%.^2^ There are molecular markers and genetic mutations that have been identified in the genomes of AML patients and cytogenetic data indicates that they are: Runx1, Flt3-ITD, MLL-PTD, NPM1, CEBPA, and ASXL1.^3^ There are about 25% of cases of AML that have mutations in the Flt3 (FMS-like tyrosine kinase-3 receptor) gene. Mutations in this gene are associated with poor outcomes.^4^ Flt3 mutations can be classified into two types: internal tandem duplications of 3 to over 100 amino acids in the juxtamembrane domain (Flt3/ITD) and point mutations in the tyrosine kinase domain (Flt3/KD).^2,4,5^ In this mini-review, we focused on these markers for the purpose of treating AML patients by inhibiting the Flt3 tyrosine kinase domain and blocking TIM-3 signaling.

## 2 FLT3 AND FLT3L

FMS-like tyrosine kinase-3 (Flt3; CD135) is a 993 amino acid single transmembrane type III receptor tyrosine kinase in the same family as macrophage colony-stimulating factor (M-CSF) receptor (M-CSFR), mast/stem cell growth factor receptor (SCFR/c-KIT/CD117), and platelet-derived growth factor receptors A and B (PDGFR-A and -B) that has one extracellular domain, five immunoglobulin folds, a juxtamembrane domain, and the cytoplasmic tyrosine kinase domain.^4,6^ Flt3 is expressed by hematopoietic stem cells,^4,6^ dendritic cells (DCs)^7^ and its signaling is crucial for hematopoiesis of CD34^∗^ hematopoietic stem cells.^8^

Flt3L is a transmembrane type I protein that is a ligand for Flt3 and belongs to the same family as SCFR and M-CSFR ligands. Five extracellular domains, a transmembrane domain and a cytosolic tail construct this protein. Contrary to Flt3 receptor, most hematopoietic and non-hematopoietic tissues express Flt3L mRNA.^9^ The CD4^∗^ T cells responsible for the hematopoietic source make Flt3L. Specifically, when CD4^∗^ T cells are stimulated with anti-CD3ε and anti-CD28 antibodies, they secrete more Flt3L than CD8+ T cells.^10^ Flt3L has three main isoforms; one of them is cleavable membrane-bound Flt3L that can be converted to soluble Flt3L. The second form lacks a transmembrane domain and is a soluble protein. Lastly, the third is not cleavable and is membrane-tethered. Each form is found in different abundances among different species; however, all are biologically active. A membrane-bound version of Flt3L is the most common type observed in humans.^6^

## 3 DCS AND ANTIGEN PRESENTING

The DC is an important antigen-presenting cell.^11^ Through acquisition, processing, and presentation of the peptide of antigen, DCs trigger immune responses and activate T lymphocytes. It is possible to develop immunotherapies based upon the biology of DCs to combat infection, cancer, and autoimmune disease. Numerous studies have shown that Flt3 plays a significant role in DC maintenance and development. Thus, Flt3 is applied to manipulate DCs to produce high-efficiency immunotherapy in vitro and in vivo.^6^ DCs can take up dead tumor cells,^12^ pathogens, apoptotic cells, and infected cells and process antigens derived from these particles into peptides, and load these peptides into major histocompatibility complex (MHC) class I and MHC class II molecules.^11^

As part of MHC class II (HLA-II) antigen presentation, exogenously derived antigens must be processed and loaded into the endosomal/lysosomal pathway. Newly synthesized HLA class II α and β heavy chains dimerize and associate with the invariant chain (Ii or CD74). The cytoplasmic tail of Ii is then specifically targeted by endosomal proteases (cathepsins) and is degraded to a small component that is called class II-associated invariant chain peptide (CLIP). CLIP is converted with exogenous peptides by HLA-DM (HLA-like chaperone molecules). After peptide exchange, the HLA class II molecules are recruited onto the plasma membrane, where it is introduced to CD4^∗^ T cells.^12^

In myeloid DCs, it has been demonstrated that TAAs (tumor-associated anti-gens) can be taken up as exogenous material (for instance, tumor cell fragments) and presented by HLA class I molecules via a process known as cross-presenting^12^ and also, recently, studies have shown that antigen-presenting cells such as DCs can process endogenous antigens on HLA class II molecules, which is known as “reverse cross-presentation.”^13^

## 4 FLT3 MUTATION AND DCS

Flt3 is expressed by all cells that differentiate into plasmacytoid DCs and conventional DCs (cDCs).^14^ Splenic cDC1 has the highest Flt3 expression, while pre-cDC1 and pre-cDC2 have the lowest.^15^ Interestingly, langerhans cells, DCs that are epidermal-resident, do not suffer from the suspension of Flt3 signaling, suggesting that their establishment and maintenance are independent of that receptor. As mentioned above, several types of DCs rely on Flt3 for development and maintenance. The Flt3 signaling pathway regulates the differentiation and mobilization of progenitor DCs, as well as the homeostatic division of cDCs in peripheral lymph nodes. DC homeostasis mediated by Flt3 is vital to normal immune system function.^6^

Aberrant Flt3 signaling in DCs through Flt3 mutations in AML patients may alter immune surveillance in cancer due to the relationship between DCs and regulatory T cells (Tregs) because DCs regulate the proliferation of Tregs in the periphery.^6^ Malignant clones of myeloid and plasmacytoid DCs have been identified in AML patients.^16^ Since approximately 60% to 80% of primary AML samples may generate DC in in vitro culture, one might consider AML to be a type of malignancy of bone marrow-derived from DC precursors. These DCs (AML-DC) are derived from the leukemic clone^17^ and may express leukemic antigens.^18^ These DCs promote tumor progression by the production of IL-10 and tolerogenic signals. In AML, since the precursor cells are myeloid, patients with the fulminant disease may have compromised myeloid function.^19^ Rather than blocking antibacterial responses, leukemic blasts suppress responses through inappropriate phagocytosis, bactericidal activity, chemotaxis, or antigen presentation.^16^ The ability to generate leukemic DC that are capable of presenting antigen in in vitro suggests that, in some cases, leukemic blasts can continue to be differentiated into antigen-presenting cells, although in vivo such differentiation has yet to be verified. If a proportion of leukemic cells matured into normal DC, the remaining leukemic blasts may directly interfere with antigen presentation to autologous T cells. Flt3-ITD mutation causes persistent and constitutive signaling of tyrosine kinase receptor that can provide survival of malignant cells and excess normal receptor-ligand interaction response but do not affect differentiation. AML is considered to be the result of both an insufficient tumor surveillance mechanism and an immune environment that allows the clone to grow.^18^

**Figure 1:**
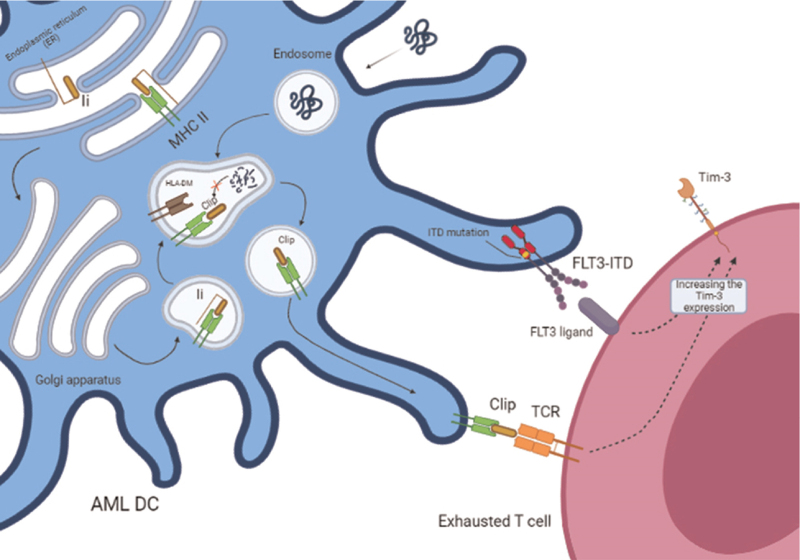
Flt3-ITD mutation, dendritic cells antigen-presenting, and TIM-3 expression in AML: MHC-II antigen presentation involves endosomal/lysosomal processing of exogenous antigens. Ii dimerizes with the newly synthesized HLA class II α and β heavy chains in ER. The cytoplasmic tail of Ii is then degraded to a small component, CLIP, by cathepsins in the endolysosome. CLIP is converted with peptide by HLA-DM, but persists on the AML-DC surface due to defective antigen presentation by Flt3-ITD. MHC-CLIP presentation to TCR and also the connection of Flt3 receptor with Flt3-ITD mutation and Flt3L on T cells cause higher TIM-3 expression, as a marker of exhausted T cells. MHC-II, major histocompatibility complex II; Ii, invariant chain; HLA class II, human leukocyte antigen class II; ER, endoplasmic reticulum; CLIP, class II-associated invariant chain peptide; HLA-DM, human leukocyte antigen DM; AML-DC, acute myeloid leukemia dendritic cell; Flt3-ITD, FMS-like tyrosine kinase-3 internal tandem duplication; TCR, T-cell receptor; Flt3, FMS-like tyrosine kinase-3; Flt3L, FMS-like tyrosine kinase 3 ligand; TIM-3, T cell immunoglobulin, and mucin-domain-3. The figure was designed by http://biorender.com.

In a functional immune system, residual leukemia cells can be eliminated. Despite this, the anti-tumor immune response is often suppressed by various mechanisms currently under investigation. A number of mechanisms may contribute to the resistance of AML blasts to the immune system. Immune evasion involves decreasing HLA expression, which impairs CD4^∗^ or CD8^∗^ T-cell recognition of antigen-specific tumors. Leukemia cells can escape elimination by cytotoxic T-cells when they express lower levels of HLA class I.^20^ We previously mentioned CLIP is an invariant chain peptide that is needed for antigen presentation with HLA class II in the exogenous pathway. The CLIP protein can also be cross-presented on HLA class I.^21^ During defective antigen presentation, CLIP remains attached to HLA molecules and is exposed on the cell surface. The worst outcome of AML is associated with leukemia blasts that present CLIP on their surfaces. Furthermore, the CLIP exposition was also higher in patients with Flt3-ITD mutation (Fig. 1).^20^ Studies have found that TIM-3 expressed not only on CD8+ T cells and other lymphocyte subsets but also on dendritic cells, monocytes and mediate immune suppression through different mechanisms.^22^ Transcript levels of TIM-3 correlated with the level of TIM-3 protein, CLIP, and PD-L1 (programmed death-ligand 1), indicating a complex immunoresistant phenotype. Flt3-ITD was associated with higher positivity of CD4+ T-cells for TIM-3.^20^ Therefore, TIM-3 expression on dendritic cells is also possibly related to the Flt3-ITD mutation on DCs. However, there is a need for future research in this field and prove this correlation.

## 5 FLT3-ITD AND T CELLS

T cell immunoglobulin and mucin-domain containing-3 (TIM-3) acts as inhibitory costimulatory molecules in T cells.^22^ AML blasts present TIM-3 as a marker of exhausted T cells.^20,23^ We only partially understand how it functions in leukemia cells and it is likely complicated.^20^ In the bone marrow and peripheral blood of AML patients, exhausted T cells have different phenotypes between subsets.^23^ Li et al have reported that CD4^∗^ and CD8^∗^ T cells from recently diagnosed AML patients express more TIM-3 than those from normal controls. Not only T cells but also DCs and monocytes can express TIM-3.^22^ The autocrine or paracrine signaling pathways of TIM-3 promote leukemia cell proliferation and resistance to apoptosis.^24^ It is also secreted together with galectin-9, which blocks the activity of T-cells, contributing to immune evasion.^22,23,25^ The secreted form of TIM-3 may exert a distant inhibitory effect on immune cells away from the leukemia cells. Because the TIM-3 gene is located on chromosome 5, the aberrant TIM-3 mRNA level might be caused by chromosome 5 duplication seen in the karyotype of an AML patient. The TIM-3 transcript appears to be correlated with CLIP expression, this suggests that immune escape mechanisms are frequently activated simultaneously.^20^ Additionally, Flt3-ITD is linked to a higher positivity of CD4^∗^ T cells for TIM-3, an immunosuppressive molecule.^22,23^ Kuželová et al showed that high levels of TIM-3 and CLIP are correlated with worse prognosis. To accomplish this, in immune resistant cells, inhibitory receptors are expressed, antigen presentation is reduced, as well as inhibitory molecules such as TIM3 are secreted (Fig. 1).^20^

## 6 THERAPEUTIC APPROACHES

In recent days, there have been new therapies for treating AML patients, including improved chemotherapy, antibodies that target immune checkpoints,^2,3^ cell cycle checkpoint inhibitors, epigenetic regulators, and microenvironment therapies.^2^

TIM-3 has been identified as an immune checkpoint target for both solid tumors and hematologic malignancies.^26^ A blockade of TIM-3 has been shown to improve the proliferation and activity of tumor antigen-specific T cells.^27^ The use of anti-TIM-3 antibodies, either alone or in combination with other drugs in clinical trials, has been found to be effective in treating a variety of cancers. Early clinical trials have been reported for three molecules as TIM-3 blockers so far: TSR-022 (Tesaro), LY3321367 (Eli Lilly and Company), and Sabatolimab (Novartis Pharmaceuticals; developed as MBG453).^26^

Flt3 TKIs (Flt3 tyrosine kinase inhibitors) have been demonstrated to inhibit the constitutive kinase activity of Flt3 mutations both in vitro and in vivo in preclinical and clinical studies. Zhu et al studied the combination of Flt3 TKIs (Gilteritinib or Sorafenib) with BCL-2 selective inhibitor (BCL-2i) that synergistically enhanced apoptosis and reduced cell proliferation in Flt3/ITD cell lines and primary AML samples.^5^

One of the potential therapeutic agents for AML is Flt3L. While it has been reported that Flt3L may cause lymphoproliferative malignancies with the described mutations of Flt3 in AML, no clinical trial has confirmed this issue when flt3L has been administered to patients.^28^ The use of retroviral vectors expressing Flt3L has shown the stimulation of the immune system in some AML murine models^29^ despite the fact that it is a controversial therapy. Therefore, further clinical trials and research are needed to determine if Flt3L could be considered as a potential treatment for AML in humans.

## 7 CONCLUSIONS

Many studies have shown that AML patients have molecular mutations such as Flt3-ITD that are evident in their karyotypes. DCs as immune cells play significant roles in priming T cells and struggling with cancers, but in AML, most DCs and myeloid precursors are malignant. As a result of Flt3-ITD mutation, DC antigen presentation capacity is reduced and T cells present exhausted phenotypes. Leukemia cells express TIM-3, which may be used as a biomarker and therapeutic target for AML. However, some clinical trials have found that blocking TIM-3 alone does not provide clinical benefit for the majority of patients with AML. As Flt3-ITD is associated with a higher expression of TIM-3 on T cells, so combining immune therapies that target both the Flt3 receptor and TIM-3 may prove more effective. Despite this, future clinical trials and research will be needed to prove the efficacy and safety of these immunotherapies. Flt3L, as the ligand of this receptor, is expressed on most hematopoietic and non-hematopoietic cells. Flt3L administration for AML remains controversial and hence, there is a need for more studies to confirm whether it can be used as a potential treatment for AML patients.
